# The effects of VIA® Instant Coffee on bench press performance: a gender comparison

**DOI:** 10.1186/1550-2783-11-S1-P13

**Published:** 2014-12-01

**Authors:** A Wise, M Frank, A Holy, S Mohney, L Lowery

**Affiliations:** 1 University of Mount Union, Alliance, Ohio, USA

## Background

An abundance of research is available concerning exercise and caffeine with men, but among women resistance trainers, it is scarce. We tested a highly caffeinated coffee product, Via® instant coffee (VIA) vs. its decaffeinated version (DCF) on men and women. We hypothesized that VIA consumption would not benefit men greater than women (p>0.05) when comparing muscle explosiveness among resistance-trained (6.4±3.7 y) college students (N=23; 21.2+3.7 y).

## Methods

After 24 hours of dietary control and caffeine abstinence, fasted subjects volunteers to perform three separate repetitions of strict Smith bench press (30% 1RM) under two conditions (VIA, DCF), with conditions separated by 48-72 hours. The peak force (FOR), peak power (POW), peak velocity (VEL), and maximum rate of force development (RFD) of the VIA trial were compared to DCF. FOR, POW, VEL, and RFD were measured via Ballistic Measurement System (BMS) linear displacement (XPV6+, Innervations, Inc., South Australia, Australia).The interaction of coffee and gender were analyzed in both absolute and relative terms via 2x2 ANOVA with repeated measures (covariance as necessary) and Tukey HSD post hoc (Statistica 12, Statsoft, Inc., Tulsa, OK). Consent to publish the results was obtained from all participants.

## Results

VIA enhanced absolute POW for men (VIA 674.9 ±167.2 W vs. DCF 622.9±154.1 W; p<0.001) and for women (VIA 274.3±49.6 W vs. DCF 249.1±44.7 W; p=0.039); absolute VEL for men (VIA 1.22±0.15 m/s vs. DCF 1.14±0.15 m/s; p=0.002) and for women (VIA 1.16±0.09 m/s vs. DCF 1.05±0.06 m/s; p<0.001); and RFD for men (VIA 1974.2±657.6 N/s vs. DCF 1788.6±529.4 N/s; p=0.001) but not for women (VIA 746.3±205.5 N/s vs. DCF 701.4±193.6 N/s; p=0.729). Further, absolute enhancement was greater for men in POW (p=0.037) and in RFD (p=0.028). No gender differences persisted when enhancement was expressed as percent increases over baseline (DCF) or after statistically adjusting for body mass.

## Conclusions

These data support the hypothesis that men and women benefit similarly (7-10%) after ingesting two servings of Via instant coffee approximately one hour before Smith bench press exercise.

